# Supplementary material for: Combined Transarterial Chemoembolization and Radiofrequency Ablation for Hepatocellular Carcinoma Infeasible for Ultrasound-Guided Percutaneous Radiofrequency Ablation: A Comparative Study with General Ultrasound-Guided Radiofrequency Ablation Outcomes
Source: Cancers (Basel). 2023 Oct 28;15(21):5193. doi: 10.3390/cancers15215193 (PMC10650828; doi:10.3390/cancers15215193)
Supplement: Supplementary file 1 [file cancers-15-05193-s001.zip › cancers-2617924-supplementary.pdf]

Table S1 Formulas for the albumin-bilirubin score, aspartate aminotransferase/platelet ratio index, "Model for tumor recurrence after living donor liver transplantation" (MoRAL) score, and high risk of microvascular invasion

ALBI score	[log 10 bilirubin (μmol/L) × 0.66] + [albumin (g/L) × -0.085]
APRI	[AST (IU/L) / upper limit normal of AST/PLT (× 10^9/L)] × 100
MoRAL score	11×√[PIVKA-II] +2×√ [AFP]

ALBI, albumin-bilirubin; APRI, aspartate aminotransferase/platelet ratio index; MoRAL, Model for tumor recurrence after living donor liver transplantation.

Table S2 Scoring system to predict high risk of microvascular invasion

Serum alpha-fetoprotein level (ng/mL)	Points
<15	0
≥15	1
Serum protein induced by vitamin K absence-II level (mAU/mL)	Points
<48	0
≥48	1
Peritumoral parenchymal enhancement on arterial phase	Points
(-)	0
(+)	1.5
Peritumoral hypointensity on hepatobiliary phase	Points
(-)	0
(+)	2.5
MVI risk group	Total points
MVI-high risk group	≥ 3.5
MVI-low risk group	< 3.5

The MVI prediction score model was calculated by adding the individual points for each of the four factors: α-FP, PIVKA-II, arterial peritumoral enhancement, and peritumoral hypointensity on HBP [1]. This model for predicting the presence of MVI was externally validated with an area under the receiver operating characteristic curve (AUC) of 0.82 (95% confidence interval [CI]:0.74–0.90), and its sensitivity, specificity, and accuracy were 65.2%, 85.9%, and 81.2%, respectively. In this model, one point is given to α-FP if ≥15 ng/mL, one point to PIVKA-II if ≥48 mAU/mL, 1.5 points if arterial peritumoral enhancement is present, and 2.5 points if peritumoral hypointensity on HBP is present. If the total number of points was ≥3.5, it was regarded as MVI-high risk. The predictive presence of MVI could be estimated based on the total MVI score using the results of the previous study.

AFP, alpha-fetoprotein; PIVKA-II, protein induced by vitamin K absence-II; HBP, hepatobiliary phase; MVI, microvascular invasion.

Table S3 Balance check before and after propensity-score matching for matched variables.

	Before matching TACE+RFA (*n* = 24), RFA (*n* = 371)		After matching TACE+RFA (*n* = 21), RFA (*n* = 42)	
	**SMD**	***p***	**SMD**	***p***
*Total bilirubin, mg/dL	0.357	0.123	0.166	0.591
*Prothrombin time, INR	0.376	0.092	–0.090	0.768
*Platelet count, × 10^9/L	–0.357	0.102	0.017	0.948
*log(AFP)	–0.498	0.001	–0.131	0.727
*Sex	–0.494	0.006	0.000	1.000
*Cause of liver disease (HBV)	–0.533	0.026	–0.047	0.865
*Cause of liver disease (HCV)	0.291	0.230	0.055	0.857
*Cause of liver disease (alcohol)	0.415	0.102	–0.066	0.792
*Cause of liver disease (others)	–0.045	0.827	0.109	0.686
*Periportal vein tumor location	0.452	0.089	–0.066	0.816
*Washout appearance (on PVP)	–0.386	0.073	0.148	0.610
*Enhancing capsule	–0.459	0.025	–0.178	0.560
*LR-3	0.649	0.008	–0.047	0.873
*LR-4	0.043	0.846	–0.118	0.686
*LR-5	–0.419	0.045	0.109	0.686
*LR-M	–0.410	0.011	0.109	0.686
*Non-smooth tumor margin	–0.519	0.004	0.079	0.792
Age	–0.120	0.588	–0.261	0.424
Albumin, g/dL	–0.304	0.159	0.076	0.812
ALBI grade 1	–0.203	0.359	0.191	0.508
ALBI grade 2	0.238	0.287	–0.096	0.742
AST, U/L	-0.141	0.299	–0.269	0.483
APRI	0.195	0.425	0.081	0.722
Child–Pugh classification grade	–0.123	0.521	–0.436	0.153
PIVKA-II, mAU/mL	0.177	0.409	–0.085	0.828
MoRAL score (>68)	–0.108	0.609	0.049	0.865
Tumor size (cm)	0.074	0.743	0.142	0.589
Perihepatic vein tumor location	–0.191	0.294	–0.327	0.425
Non-subphrenic subcapsular location	0.100	0.651	0.000	1.000
Subphrenic location	0.178	0.438	0.436	0.096
Non-subcapsular location	–0.224	0.310	–0.376	0.203
No non-rim APHE	0.203	0.416	–0.109	0.755
Non-rim APHE	0.044	0.833	–0.079	0.792
Rim APHE	–0.243	0.165	0.218	0.318
Peripheral washout appearance	–0.147	0.045	0.000	1.000
Progressive centripetal enhancement	–0.269	0.000	0.000	1.000
Transitional phase targetoid appearance	–0.223	0.003	0.000	1.000
HBP targetoid appearance	–0.258	0.000	0.000	1.000
DWI targetoid appearance	–0.153	0.413	0.109	0.686
Non-targetoid LR-M features	–0.410	0.011	0.109	0.686
Arterial peritumoral hyperenhancement	–0.335	0.068	–0.546	0.178
HBP peritumoral hypointensity	–0.172	0.350	–0.109	0.755
Signal intensity of tumor on HBP	0.282	0.268	–0.133	0.708
MVI-high risk	–0.172	0.350	0.000	1.000

Standardized mean differences indicate very small differences for values less than 0.10; 0.1-0.3, small differences; 0.3-0.5, moderate differences; and values higher than 0.5, large differences.

INR, international normalized ratio; AFP, alpha-fetoprotein; HBV, hepatitis B virus; HCV, hepatitis C virus; PVP, portal venous phase; LR, LI-RADS, Liver Imaging Reporting and Data System category; ALBI grade, albumin-bilirubin grade; AST, aspartate aminotransferase; APRI, AST/platelet ratio index; PIVKA-II, protein induced by vitamin K absence-II; MoRAL score, Model for Tumor Recurrence After Living Donor Liver Transplantation

score; APHE, arterial phase hyperenhancement; DWI, diffusion weighted images; HBP, hepatobiliary phase; MVI, microvascular invasion; SMD, standard mean difference; TACE, transarterial chemoembolization; RFA, radiofrequency ablation.

* Variables used for propensity score matching.

Table S4 Inter-reader agreement of the imaging findings

Variables	Kappa	95% CI
Non-rim arterial hyperenhancement	0.834	0.704–0.964
Washout appearance	0.771	0.710–0.832
Enhancing capsule	0.642	0.566–0.717
LR-M	0.758	0.681–0.835
Peri-tumoral enhancement	0.661	0.570–0.753
Non-smooth margin	0.650	0.566–0.734
Peritumoral hypointensity	0.682	0.557–0.807
Low SI on HBP (reference=iso/high)	0.859	0.736–0.981

A κ value of 0.01–0.20 was interpreted as slight, 0.21–0.40 as fair, 0.41–0.60 as moderate, 0.61–0.80 as substantial, and 0.81–1.00 as excellent agreement

REFERENCES

1. Lee, S.; Kang, T.W.; Song, K.D.; Lee, M.W.; Rhim, H.; Lim, H.K.; Kim, S.Y.; Sinn, D.H.; Kim, J.M.; Kim, K.; et al. Effect of Microvascular Invasion Risk on Early Recurrence of Hepatocellular Carcinoma After Surgery and Radiofrequency Ablation. *Ann. Surg.* **2021**, *273*, 564-571.
